# Preparation of hydrophilic nanofiltration membranes for removal of pharmaceuticals from water

**DOI:** 10.1186/s40201-015-0201-3

**Published:** 2015-05-13

**Authors:** Maryam Omidvar, Mohammad Soltanieh, Seyed Mahmoud Mousavi, Ehsan Saljoughi, Ahmad Moarefian, Hoda Saffaran

**Affiliations:** Department of Chemical Engineering, Science and Research Branch, Islamic Azad University, Tehran, Iran; Department of Chemical Engineering, Faculty of Engineering, Ferdowsi University of Mashhad, Mashhad, Iran; Department of Chemical Engineering, Quchan Branch, Islamic Azad University, Quchan, Iran

**Keywords:** Nanofiltration, Polyethersulfone, Hydrophilicity, Brij58, Pharmaceuticals

## Abstract

Asymmetric polyethersulfone (PES) nanofiltration membranes were prepared via phase inversion technique. PES polymer, Brij 58 as surfactant additive, polyvinylpyrrolidone (PVP) as pore former and 1-methyl-2-pyrrolidone (NMP) as solvent were used in preparation of the casting solutions. Distillated water was used as the gelation media. The scanning electron microscopy (SEM) images and measurements of contact angle (CA) and zeta potential were used to characterize the prepared membranes. Also performance of the membranes was examined by determining the pure water flux (PWF) and pharmaceuticals rejection. The addition of Brij 58 to the casting solution resulted in formation of the membranes with higher thickness and more porous structure in the sublayer in comparison with the net PES membrane. The surface hydrophilicity of the membranes was remarkably enhanced via the presence of Brij 58 in the casting solution, so that, the contact angel diminished from 74.7° to 28.3° with adding 6 wt. % of Brij 58 to the casting solution. The addition of Brij 58 to the casting solution resulted in formation of the membranes with superior PWF and higher rejection of amoxicillin and ceftriaxone in comparison with the pure PES membrane.

## Introduction

PES is a commercially available, thermally stable polymer, which is used in high-performance applications due to its toughness, good thermal resistance and chemical inertness [1]. As a result, PES is one of the most important polymeric materials and is widely used in separation fields [2, 3]. Though PES and PES-based membranes have been broadly applied in separation processes, they have disadvantages. The main disadvantage of the PES membranes is related to their relatively hydrophobic character [2]. Their hydrophobicity leads to a low membrane flux and poor anti-fouling properties, which have a great impact on PES membrane application and useful life [4, 5]. Membrane fouling is a common serious problem in water treatment and desalination plants employing nanofiltration (NF) and reverse osmosis (RO) membranes [6, 7]. Membrane fouling reduces membrane performance, increases operating costs, and shortens membrane life [8–10].

A general method to suppress membrane fouling, especially irreversible fouling is to inhibit natural organic matter adsorption on the membrane surface by increasing hydrophilicity of the membrane surface [8]. Many investigations have revealed that increasing the membrane surface hydrophilicity can effectively reduce the membrane fouling [9, 11]. Therefore, efforts have focused on increasing PES hydrophilicity by chemical or physical modifications such as UV irradiation [12], addition of additive [9, 13–15], plasma treatment [16, 17], and so on. Addition of surfactant additives to the casting solutions can influence morphology and performance of membranes. Some researchers studied the effects of surfactant additives on the morphology and performance of polyethersulfone ultrafiltration membranes [11, 15].

Human and veterinary pharmaceuticals have become a class of emerging environmental contaminants due to their potential undesirable effects on human health and aquatic ecosystems [18, 19]. Antibiotics are among the most commonly detected pharmaceuticals in the aquatic environment because their antibacterial nature prevents effective removal in sewage treatment plants [20].

A wide range of methodologies can be employed for rejection of different pharmaceuticals, for example, advanced oxidation process [21, 22], electrochemical removal process [18, 20], ozonation [23–25], nanofiltration [26–28] and membrane bioreactor [29, 30]. Depending on contaminant concentration in the effluent and the process cost, different methods can be chosen. Membrane filtration processes of RO and NF have been shown to have a greater ability to reject pharmaceuticals from aqueous matrices [31].

NF membranes may effectively reject antibiotics due to the membrane pore size and the compound characteristics such as low molecular weight and possible charge effects. There are several studies reported using NF as a tool for removal of pharmaceutical substances such as antibiotics. Zazouli *et al*. [32] studied the performance of two types of commercial NF membranes (SR2 & SR3) for removal aquatic pharmaceutical residual. They investigated the effect of pH, ionic strength, transmembrane pressure and natural organic material (NOM) on the drug rejection and permeate flux. The highest rejection was observed for tetracycline i.e. 75-95 % for SR2 and 95-100 % for SR3. Shah *et al*. [26] studied the mechanism of antibiotic removal by three types of commercial NF membranes of varying tightness. It was found that antibiotic rejection varies with both pH and membrane tightness. Wang and Chung [33] used two types of commercial NF membrane (NADIR N30F and NF PES 10) for separation of cephalexin. They through adjusting the pH of aqueous solution found, the separation of cephalexin can be effectively manipulated up to 98 % and 88 % for N30F and NF PES 10, respectively. N30F membrane showed higher rejection for cephalexin due to its smaller pores and larger charge density than NF PES 10 membrane. Koyuncu *et al*. [34] investigated the effect of solution chemistry, organic matter and salinity on the rejection of tetracycline’s and sulfonamide, and their adsorption on membrane of NF 200. Almost 80 % of chlortetracycline was adsorbed on the membrane surface compared with 50 % for doxcycline.

There is no report to investigate the effects of addition of Brij 58 on morphology and properties of PES nanofiltration membranes and their performance in the removal of antibiotics from aqueous solutions. Therefore, the main objective of this study is to investigate the effect of Brij 58 concentration as a surfactant additive on the PES nanofiltration membranes and evaluation of ability of the modified membranes for rejection of two antibiotics i.e. amoxicillin, as top-priority human and veterinary pharmaceutical, and ceftriaxone from water.

## Materials and methods

### Materials

Polyethersulfone (Ultrason E6020P, MW = 58,000 g/mol) supplied from BASF company was employed as basis polymer of the membranes. N-methyl-2-pyrrolidone (*>*99.5 %) and polyvinylpyrrolidone (PVP K 40) purchased from Merck (Germany) were used as solvent and pore former, respectively. Surfactant additive, Brij 58 (polyethylene glycol hexadecyl ether, C_56_H_114_O_21_) with the hydrophilic-lipophilic balance (HLB) =16 was bought from Sigma–Aldrich. Distilled water was used as nonsolvent. Amoxicillin (C_16_H_19_N_3_O_5_S) and ceftriaxone (C_18_H_18_N_8_O_7_S_3_) were procured from Dana pharmaceutical company (Tabriz, Iran). Table 1 summarizes characteristics of these pharmaceuticals. N, N-dimethyl-*p*-phenylenediamine, potassium hexacynoferrate (III), iron (III) nitrate.9H_2_O, NH_3_ and NaOH were purchased from Merck.

**Table 1:** Characteristics of the selected pharmaceuticals

Molecular Structure	M_w_, g mol^−1^	Dissociation constants (Pk_a_)
Amoxicillin	365.4	2.4, 7.31, 9.53 [57]
Ceftriaxone	554.58	3, 3.2, 4.1 [58]

### Preparation of membranes

Homogeneous solutions containing PES polymer, NMP solvent, PVP as invariable additive (pore former) and the specific amount of Brij 58 surfactant (0–8 wt. %) as variant additive were prepared by stirring (200 rpm) for 12 h at ambient temperature (25 ± 2 °C). The dope solutions were held at ambient temperature for almost 12 h to remove air bubbles. The solutions were cast onto a glass plate with a film applicator. Then they were immersed in distilled water bath (25 ° C) for 12 h to complete the phase separation where exchange between the solvent and nonsolvent was induced. For drying the membranes, they were kept between two sheets of filter paper for 24 h [11, 15]. Composition of the casting solutions are shown in Table 2.

**Table 2:** Composition of the casting solutions and zeta potential of the membranes

Membrane	PES (wt. %)	PVP (wt. %)	Brij 58 (wt. %)	Zeta Potential at pH 5 (mV)
M1	21	2	0	5.59-
M2	21	2	2	8.04-
M3	21	2	4	9.85-
M4	21	2	6	12.2-
M5	21	2	8	13.6-

### Characterization of nanofiltration membranes

In order to characterize the prepared nanofiltration membranes, scanning electron microscopy (SEM) and measurement apparatuses of contact angle and zeta potential were employed.

#### Scanning electron microscopy (SEM)

Structure of the prepared membranes was examined by a scanning electron microscope (KYKY-EM 3200, China). For preparing the images of the cross section, the membranes were first frozen in liquid nitrogen and then fractured. After sputtering with gold, they were viewed with the microscope at 25 kV.

#### Zeta potential measurement

Membrane surface charge has a significant effect on performance of the membrane filtration process [35]. To determine the membrane surface charge, the zeta potential was determined from streaming potential measurements by Electro Kinetic Analyzer (EKA 1.00, Anton-Paar, Swiss) equipped with a plated sample cell. The measurements were carried out at 25 °C in KCl solution (0.001 M, pH 5) with poly (methyl methacrylate) (PMMA) reference plate.

#### Contact angle measurement

Membrane hydrophilicity was quantified by measuring the contact angle between the membrane surface and water. The contact angles were measured using a contact angle measuring instrument [G10, KRUSS, Germany]. The contact angle values of each sample were obtained at four various positions of the sample and then the average value was recorded.

#### Nanofiltration experiments

All experiments were carried out at room temperature (25 ± 2 °C) and transmembrane pressure (TMP) of 10 bar using a cross flow nanofiltration set up (33) with effective membrane surface area of 57 cm^2^ in batch mode.

The membranes performance was characterized by pure water flux (PWF) and antibiotics rejection. The pure water flux was calculated by the following equation [36]:

1 $$ \mathrm{P}\mathrm{W}\mathrm{F}=\mathrm{Q}/\mathrm{A}.\varDelta \mathrm{t} $$

Where Q is the permeate quantity (l), *A* is the effective membrane surface area (m^2^) and Δt is the sampling time (h).

After pure water filtration, the feed reservoir was emptied and refilled with the feed solution in order to its filtration. The feed solutions were prepared by dissolving the specific amounts of amoxicillin or ceftriaxone in distilled water. In the experiments, the feed solutions contained 20 mg/l amoxicillin or ceftriaxone.

The solute rejection was calculated using Eq. (2) [36]:

2 $$ \mathrm{R}\left(\%\right)=\left(1\hbox{--} {\mathrm{C}}_{\mathrm{p}}/{\mathrm{C}}_{\mathrm{F}}\right)\times 100 $$

Where C_P_ and C_F_ are the concentrations of the solute in the permeate and feed solutions, respectively. In order to calculate the concentration of the antibiotics, their absorbance was measured in the appropriate wavelength [37, 38] using UV–Vis spectrophotometer (T60, China).

## Results and Discussion

### Effect of Brij 58 on morphology of the membranes

**Fig. 1:**
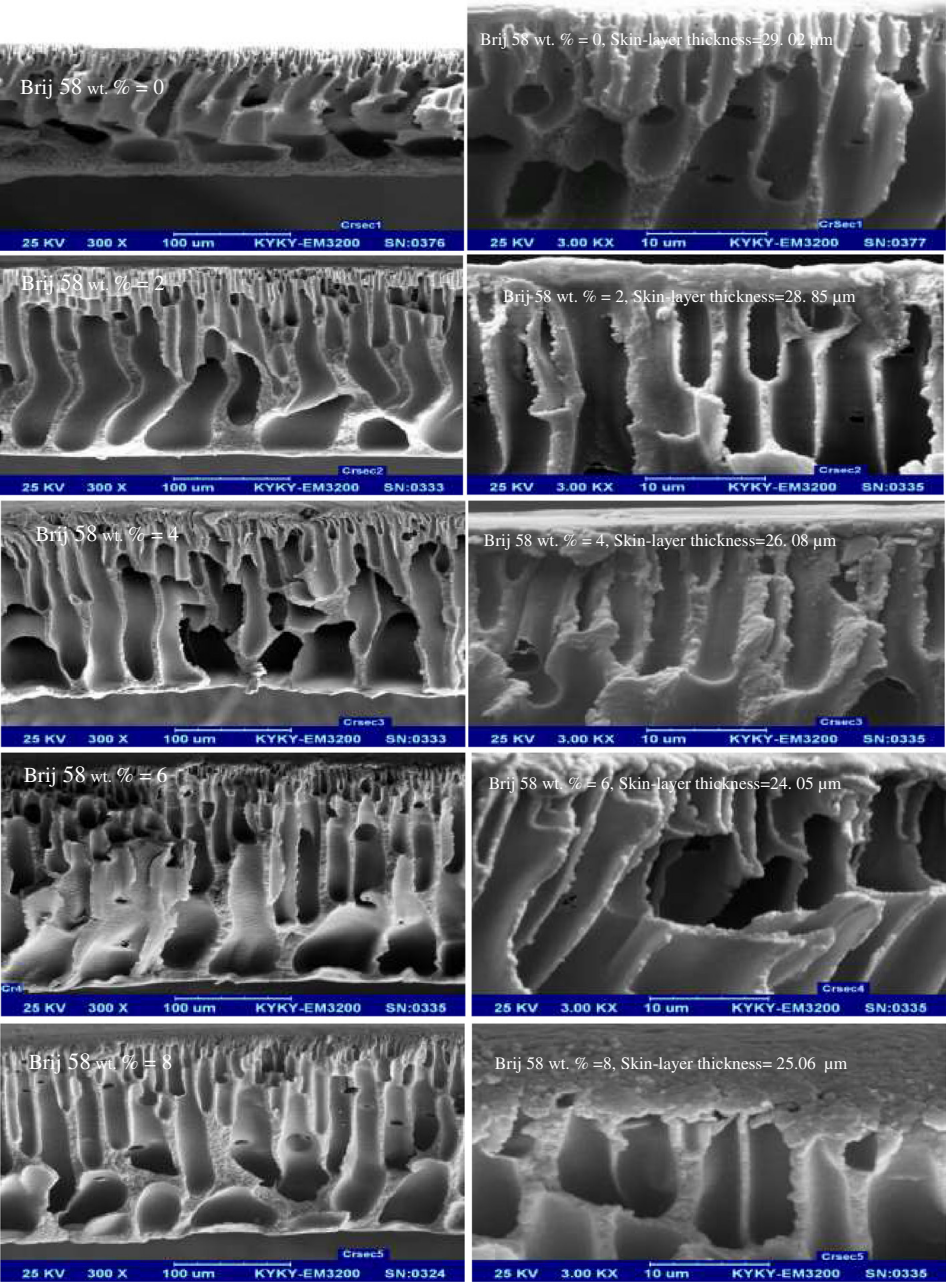
SEM cross- section images of the prepared membranes with two magnification

In order to understand the influence of Brij 58 surfactant on the membrane structure, cross-section of the membranes was observed using SEM. The cross-sectional images with two different magnifications are shown in Fig. 1.

All of the membranes exhibit asymmetric morphology consisting of a dense top-layer and a porous sublayer. Addition of Brij 58 resulted in the membranes with thinner skin-layer and more porous sublayer in comparison with the net PES membrane; while addition of 8 wt. % Brij 58 resulted in formation of a less porous structure with thicker skin-layer in comparison with the membrane prepared with 6 wt. % of Brij 58. The mentioned changes on the membranes morphology can be attributed to the interactions between the components in the casting solution. Addition of a hydrophilic additive with nonsolvent properties reduces the thermodynamic stability of the dope system [36, 39–43]. In addition, hydrophilic nature of the additive accelerates the in-diffusion rate of nonsolvent (water) during membrane formation [36, 39, 41, 43–48]. It is likely that both the reduction in thermodynamic stability and increase in nonsolvent in-diffusion rate promote instantaneous demixing, which enhances the macrovoid formation [39]. On the other hands, addition of a hydrophilic additive into the casting solution leads to the formation of complexes between additive and polymer resulting in a reduction of the interactions between polymer chains. Therefore, the penetration of nonsolvent into the chain spaces can be increased. The evident result of this phenomenon is the facilitation of instantaneous demixing in the coagulation bath and consequently the formation of membranes with higher porosity [45–47, 49–51]. From another point of view, increasing the concentration of hydrophilic additive in the casting solution results in the viscosity increase which affects thickness of the top-layer and compactness of the prepared membranes [36, 39, 45, 47, 48, 52]. Viscosity of casting solution is an important parameter for determining the phase inversion rate and membrane morphology. The casting solutions containing an additive exhibit different rheological properties [53].

Increasing the concentration of Brij 58, a hydrophilic additive, from 0 to 6 wt % into the casting solution leads to the formation of complexes between the additive and polymer resulting in a reduction in the interactions between polymer chains. Moreover, this additive influences the penetration rate of nonsolvent (water) and increases the demixing rate of the casting solution. Therefore, as aforementioned the skin thickness decreases and the porosity of the sublayer in the membranes increases. In these concentrations, the casting solution viscosity is not dominant factor for determining the membrane morphology. It seems that at higher concentration of Brij 58 i.e. 8 wt. %, the casting solution viscosity is effective factor for controlling the membrane morphology and can effectively reduce the phase inversion rate. The evident result of this phenomenon is the formation of denser membrane with thicker top-layer in comparison with the membrane containing 6 wt. % of Brij 58. Similar results about the morphology were observed by Saljoughi et al. regarding the preparation of PSF/IGEPAL NF membranes [36].

### Effect of Brij 58 on contact angle of the membranes

**Fig. 2:**
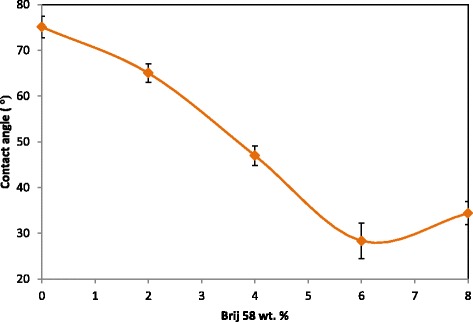
Contact angle of the prepared membranes

Figure 2 shows the effect of addition of Brij 58, as a hydrophilic surfactant, on the contact angle and in other words wettability of the membranes. As shown, the membranes prepared with addition of Brij 58 present higher hydrophilicity (lower water contact angle) in comparison with the pure PES membrane. The highest water contact angle and in other words, the highest hydrophobicity belong to the pure PES membrane. Water contact angle of the PES membranes remarkabely decreased from 74.7° to 28.3° after adding 6 wt. % of Brij 58 and then slightly increased with adding 8 wt. % of Brij 58. Higher hydrophilicity of the PES/Brij 58 membranes in comparison with the pure PES membrane can be related to hydrophilic nature of Brij 58 and the accumulation of this surfactant on the surface of the membranes. Higher contact angle of the membrane prepared from 8 wt. % of Brij 58 in comparison with the membrane prepared from 6 wt. % of Brij 58 can be related to difference in the membrane surface porosity. In fact, lower porosity of membrane surface can increase the contact angle [13].

### Effect of Brij 58 on PWF

**Fig. 3:**
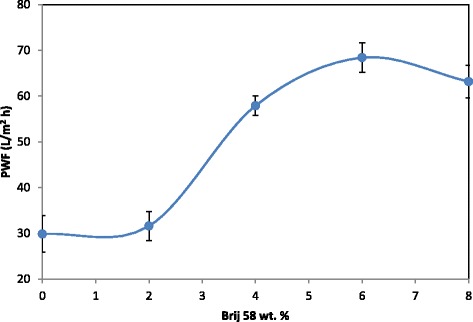
PWF of the prepared membranes

Figure 3 reveals the effect of Brij 58 concentration on PWF of the prepared membranes at TMP of 10 bar. As shown, PWF of all the PES/Brij 58 membranes increased in comparison with that of the pure PES membrane. For example, PWF of the membranes increased from 28.94 l/m2 h to 68.42 l/m^2^ h after adding 6 wt. % of Brij58 and then slightly decreased with addition of 8 wt. % Brij 58 to the casting solutions. The above trend confirms the results observed from the aforementioned SEM images. In fact, the membranes with higher porosity and thinner dense top layer presented higher PWF. It is evident that there is a direct relationship between the porosity and permeability.

### Effect of Brij 58 on rejection of antibiotics

**Fig. 4:**
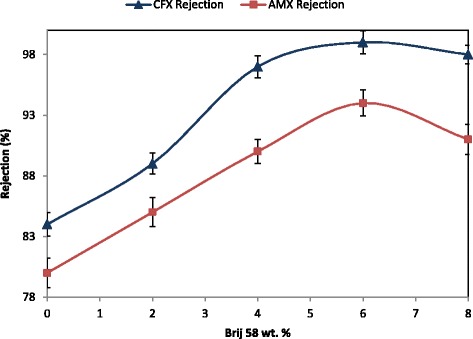
Amoxicillin (AMX) (20 mg/l, pH =5.27) and ceftriaxone (CFX) (20 mg/l, pH =5.07) rejection of the prepared membranes

The results of rejection of amoxicillin and ceftriaxone molecules obtained by utilizing the prepared membranes are illustrated in Fig. 4 As observed, all the PES/Brij 58 membranes revealed higher rejection in comparison with the pure PES membrane. The initial increase in Brij 58 concentrations up to 6 wt. % resulted in increasing the amoxicillin and ceftriaxone rejection, however, further increase in Brij58 concentration up to 8 wt. %, resulted in decreasing the rejection of the mentioned solutes. Also, for all the membranes, rejection of ceftriaxone molecules was higher than that of amoxicillin molecules, so that, the highest rejection (99.5 %) was obtained for ceftriaxone molecules using the PES membrane prepared with adding 6 wt. % of Brij 58 in the casting solution.

Any variation on the performance of the prepared membranes after adding Brij58 into the casting solution originates from the changes on morphology and other properties of the membrane. The retention behavior of organic molecules by NF membranes can be attributed to some mechanisms including size exclusion (steric hindrance), electrostatic charge repulsion, and adsorption on the membrane surface [45–47, 49]. These mechanisms are related to the membrane and solute properties as well as solution conditions [49]. Because of hydrophilic property of amoxicillin and ceftriaxone [54, 55], they are not mostly adsorbed on the membrane surface. Consequently, the rejection of the mentioned solutes can only occur due to either steric effects for uncharged solutes or combined steric and electrostatic effects for charged solutes.

During the filtration process, NF membranes are charged, which is mostly due to the ionic dissociation or protonation of functional groups on the membrane surface at different solution conditions [46]. When the solute is charged and has the same charge as the membrane surface charge, the electrostatic charge repulsion forces do not allow it to get close the surface and eventually, this charge repulsion is the dominant mechanism of separation of charged organic compounds [49]. Besides the importance of the influence of solute and membrane properties on the separation efficiency as mentioned above, feed pH has also some effects on the organic solute rejection which is due to its effect on both membrane surface and organic solute charge [49]. Amoxicillin and ceftriaxone reveal different properties at various solution pHs due to their acid dissociation constants (pKa). According to Table 1, the pKa value of amoxicillin is 2.4 (COOH), 7.31 (NH_3_^+^) and 9.53 (enolic OH), and that of ceftriaxone is 3 (COOH), 3.2 (NH_3_^+^) and 4.1 (enolic OH). Consequently, in the feed solution containing 20 mg/l amoxicillin with pH =5.27, amoxicillin molecules become neutral [30, 51] and in the feed solution containing 20 mg/l ceftriaxone with pH =5.07, ceftriaxone molecules possess negative charge [56].

One of the most important factors which significantly influences on the retention of charged solutes is the charge of membrane surface (zeta potential value). According to the zeta potential measurements presented in Table 2, by addition of Brij58, the negative charge on the membrane surface is increased. On the other hand, ceftriaxone molecules are negatively charged. As mentioned before, the electrostatic charge repulsion between negatively charged solutes and membrane surface can intensify the rejection of the mentioned charged solutes. Therefore, regarding the separation of charged solutes such as ceftriaxone in this study, increasing the negative charge of the membrane surface, as a result of addition of Brij 58, improves the separation performance of the PES/Brij 58 membranes.

The morphological changes induced on the membranes after addition of Brij 58 should also be considered as another important factor influencing the separation performance of the membranes. Amoxicillin and ceftriaxone molecules are too large in comparison with water molecules. According to Fig. 1, the membrane prepared without Brij 58 additive is denser in comparison with the other membranes and comprises thicker dense top layer. Thus, the resistance of this membrane against the permeation of both water and antibiotics molecules is noticeable. As mentioned before and according to SEM images, the increase in Brij 58 concentration up to 6 wt. % results in the formation of more porous structures with thinner dense top layer which consequently facilitates the transmission of both water and antibiotics molecules. The increase in amoxicillin and ceftriaxone rejection can be related to the moderate increase in the porosity that results in the moderate reduction of the resistance against the feed permeation. This moderate change of morphology can be more effective on the transmission of tiny components similar to water molecules in comparison with the large components such as amoxicillin or ceftriaxone molecules. This can lead to the reduction of amoxicillin and ceftriaxone concentrations in the permeate stream and consequently higher rejection of these solutes. Similar results and discussion were presented by Saljoughi et al. [36] regarding the separation of arsenic by the NF polysulfone membrane. As mentioned before, further increase in the Brij 58 concentration from 6 wt. % to 8 wt. %, results in the formation of denser structure and according to the above description, slightly decreases the rejection value.

Higher rejection of ceftriaxone in comparison with that of amoxicillin can be attributed and interpreted by:

As mention before amoxicillin molecules are neutral whereas ceftriaxone molecules are negatively charged. Thus, the electrostatic charge repulsion between ceftriaxone and membrane surface intensifies the rejection of this solute in comparison with that of amoxicillin.

Molecular weight of ceftriaxone is greater than that of amoxicillin according to the data of Table 1. This can prevent easy transmission of ceftriaxone in comparison with that of amoxicillin.

## Conclusion

Modification of PES nanofiltration membranes was carried out by the addition of different values of Brij 58 surfactant additive to the casting solution. The prepared membranes after addition of Brij 58 revealed the structures with thinner skin-layer and higher sublayer porosity in comparison with the pure PES membrane. The surface hydrophilicity of the nanofiltration membranes was significantly enhanced via the presence of Brij 58 in the casting solution. The results indicated that the nanofiltration membranes with higher PWF were prepared by adding Brij 58 to the casting solution. PES/Brij 58 membranes presented remarkably rejections of about 94 % and 99 % for amoxicillin and ceftriaxone, respectively.
